# Effective Modulation of Optical and Photoelectrical Properties of SnS_2_ Hexagonal Nanoflakes via Zn Incorporation

**DOI:** 10.3390/nano9070924

**Published:** 2019-06-27

**Authors:** Ganesan Mohan Kumar, Pugazhendi Ilanchezhiyan, Hak Dong Cho, Shavkat Yuldashev, Hee Chang Jeon, Deuk Young Kim, Tae Won Kang

**Affiliations:** 1 Nano-Information Technology Academy (NITA), Dongguk University-Seoul, Seoul 04623, Korea; 2 Quantum Functional Semiconductor Research Center, Dongguk University-Seoul, Seoul 04623, Korea; 3 Division of Physics and Semiconductor Science, Dongguk University-Seoul, Seoul 04623, Korea

**Keywords:** SnS_2_ nanoflakes, semiconductor, zinc doping, photoelectronics

## Abstract

Tin sulfides are promising materials in the fields of photoelectronics and photovoltaics because of their appropriate energy bands. However, doping in SnS_2_ can improve the stability and robustness of this material in potential applications. Herein, we report the synthesis of SnS_2_ nanoflakes with Zn doping via simple hydrothermal route. The effect of doping Zn was found to display a huge influence in the structural and crystalline order of as synthesized SnS_2_. Their optical properties attest Zn doping of SnS_2_ results in reduction of the band gap which benefits strong visible-light absorption. Significantly, enhanced photoresponse was observed with respect to pristine SnS_2_. Such enhancement could result in improved electronic conductivity and sensitivity due to Zn doping at appropriate concentration. These excellent performances show that Sn_1−*x*_Zn*_x_*S_2_ nanoflakes could offer huge potential for nanoelectronics and optoelectronics device applications.

## 1. Introduction

Metal sulfides have received considerable interest due to their unique optoelectronic properties while processed at micro-nano level [1,2,3,4]. In particular, two-dimensional (2D) metal sulfides nanostructures such as nanoplates, nanoflakes and nanosheets have received much attention for their potential application in photodetectors, photovoltaic devices and light-emitting diodes [5,6,7,8,9,10,11,12,13,14,15,16,17,18,19,20]. 2D form of the material offers high specific surface area, making it advantageous for electrochemical, catalytic and photoelectrical activities. Another advantage in 2D materials is that they are more compatible and can easily be integrated into nano-microscale structures for developing new optoelectronic devices [21,22,23,24].

Meanwhile, SnS_2_ is considered as one of the promising layered materials with excellent visible light absorption and electrical properties. It possesses band gap (2.1–2.3 eV), n-type characteristics, high sensitivity and high surface activity for applications in Li-ion batteries [25], photovoltaic devices [26] and photodetector [27,28]. Variety of nanostructures such as nanoflakes, nanosheets and nanoplates through physical and chemical techniques including chemical vapor deposition, solvothermal and hydrothermal methods have been reported by several groups [29,30]. Among them, nanoflakes preparation via hydrothermal method have attracted considerable interest due to its low cost and large-scale production at low temperatures. Similarly, many efforts have also been made in controlling morphology and enhancing the photoelectrical, chemical and physical properties for improving the device performance. Moreover, dopants in semiconductor could lead to reduction in particle size, narrowing of band gap and enhance the photoelectrical properties of SnS_2_ [31]. Recently, V and Ti doped SnS_2_ was reported to be an intermediate band material for application in wider solar absorption [32,33]. Recently doping SnS_2_ with Fe resulted in room temperature ferromagnetism [34]. Similarly, in our previous work, we reported enhanced optical and electrical properties of SnS_2_ nanoflakes via Cu doping [35]. More recently, Liu et al. reported enhanced photoresponsivity in Sb doped SnS_2_ monolayer [36]. Based on the above literatures we test the ability of doping Zn ions in SnS_2_ to significantly enhance conductivity and sensitivity favorable for its performance in photoelectronics.

The present work reports on hydrothermal synthesis of Zn doped SnS_2_ nanoflakes at low temperatures. The properties of Sn_1−*x*_Zn*_x_*S_2_ nanoflakes have been intensively studied through structural, optical and photoelectrical methods. The results show that the Zn doping results in enhanced sensitivity, conductivity and efficiency of charge transfer kinetics. As a proof of concept, Sn_1−*x*_Zn*_x_*S_2_ nanoflakes were integrated into a patterned indium tin oxide (ITO) substrate (as active material) for photoelectronic device architecture. The results showcased excellent on-off ratio and photoresponse properties than that of pristine counterpart_._ Our investigations presents Zn doped SnS_2_ could be a potential candidate for future nano electronic and photoelectronic applications.

## 2. Experiment

### 2.1. Synthesis of Sn_1−x_Zn_x_S_2_ Nanoflakes

SnS_2_ and Sn_1−*x*_Zn*_x_*S_2_ nanoflakes were prepared via low cost hydrothermal route reported previously [35]. In brief, 0.1753 g SnCl_4_·5H_2_O (Tin (IV) chloride pentahydrate) and 0.15 g thioacetamide (TAA) were dissolved in 80 mL distilled water, stirred for 1 h to result in homogeneous solution. The prepared solution was transferred to 100 mL Teflon-line autoclave, sealed and heated up to 160 °C for 12 h and finally cooled to room temperature. The prepared SnS_2_ nanoflakes were then washed with ethanol and deionized water repeatedly and finally dried at 60 °C for 12 h in electric oven. For the synthesis of Sn_1−*x*_Zn*_x_*S_2_ nanoflakes, 1 and 3 mmol% of Zinc chloride was added to the precursor solution. 

### 2.2. Characterization

The morphological evolution of the sample was examined using field-emission scanning electron microscopy (FESEM, Philips, Model: XL-30, Amsterdam, The Netherland) and field-emission transmission electron microscopy (FE-TEM, JEM-2100F HR, Tokyo, Japan). The phase purity and crystal structure of SnS_2_ and Sn_0.97_Zn_0.03_S_2_ nanoflakes was inferred through X-ray diffractometer (SmartLab, Rigaku Corporation, Tokyo, Japan). The Raman measurements were performed in a micro-Raman spectrometer (DawoolAttonics, Model: Micro Raman System, Seongnam, Korea) using an excitation wavelength of 532 nm. The chemical composition of Sn_0.97_Zn_0.03_S_2_ was obtained using X-ray photoelectron spectroscopy (K-Alpha+, ThermoFisher Scientific, Waltham, MA, USA). In order to avoid charging effect, during the measurement, charge neutralization was performed with an electron flood gun (K-Alpha+, ThermoFisher Scientific, USA). The absorbance spectrum was recorded using a UV/VIS spectrophotometer (K LAB, Model: Optizen POP, Daejeon, Korea). A Keithley 617 semiconductor parameter analyzer (Tektronix*,* Beaverton, OR, USA; Model: Keithley 617) was employed to study the photo-response of the device under solar simulator (Newport, OR, USA; AM1.5) (SERIC, Model: XIL-01B50KP).

### 2.3. Device Fabrication

Initially, 2 mg of samples SnS_2_ and Sn_0.97_Zn_0.03_S_2_ were added in 10 mL methoxy-ethanol solvent separately and magnetic stirred for 30 min followed by sonication of about 30 min to form colloidal suspension. The resulting suspension was then spin casted on cleaned and patterned ITO/glass substrate at 1000 rpm and dried at 100 °C for 5 min. Several cycles of spin casting process was repeated to obtain a continuous film.

## 3. Results and Discussions

**Figure 1:**
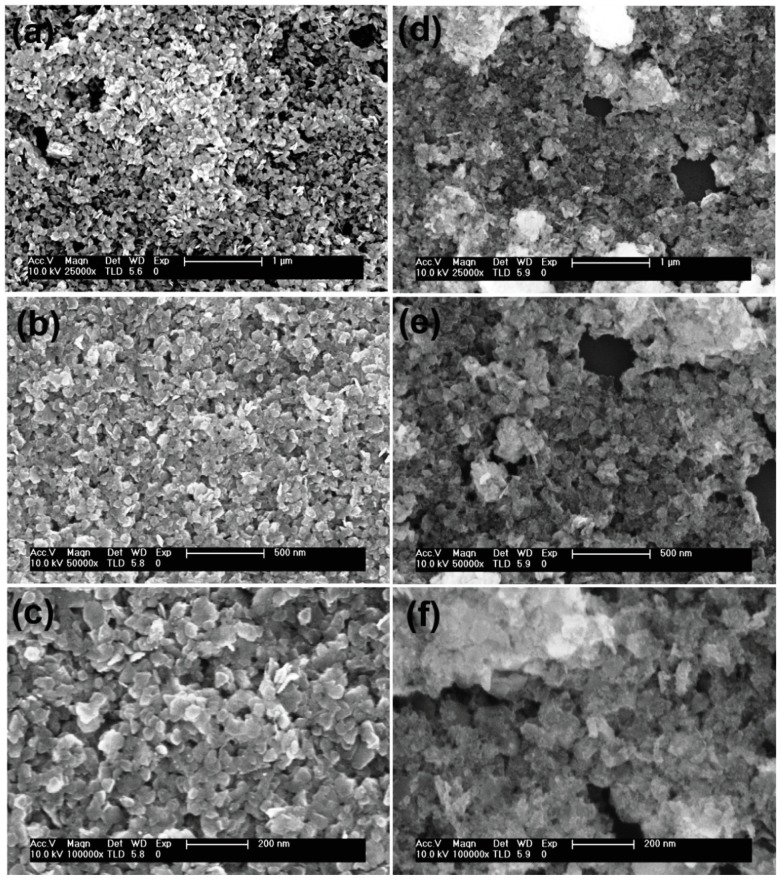
Morphological and structural characterization of SnS_2_ and Sn_0.97_Zn_0.03_S_2_ nanoflakes. (**a**–**c**) low magnification and high magnification scanning electron microscopy (SEM) image of SnS_2_; (**d**–**f**) low magnification and high magnification SEM image of Sn_0.97_Zn_0.03_S_2_ nanoflakes showing their hexagonal structure.

**Figure 2:**
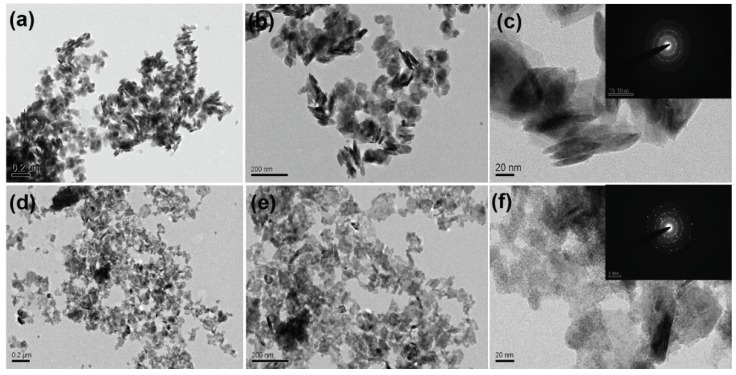
(**a**–**c**) Transmission electron microscopy (TEM) images of SnS_2_ and inset in Figure 2c shows selected area electron diffraction (SAED) pattern of SnS_2_ nanoflakes; (**d**–**f**) TEM images of a typical Sn_0.97_Zn_0.03_S_2_ nanoflakes with SAED pattern in inset of Figure 2f, revealing polycrystalline structure.

**Figure 3:**
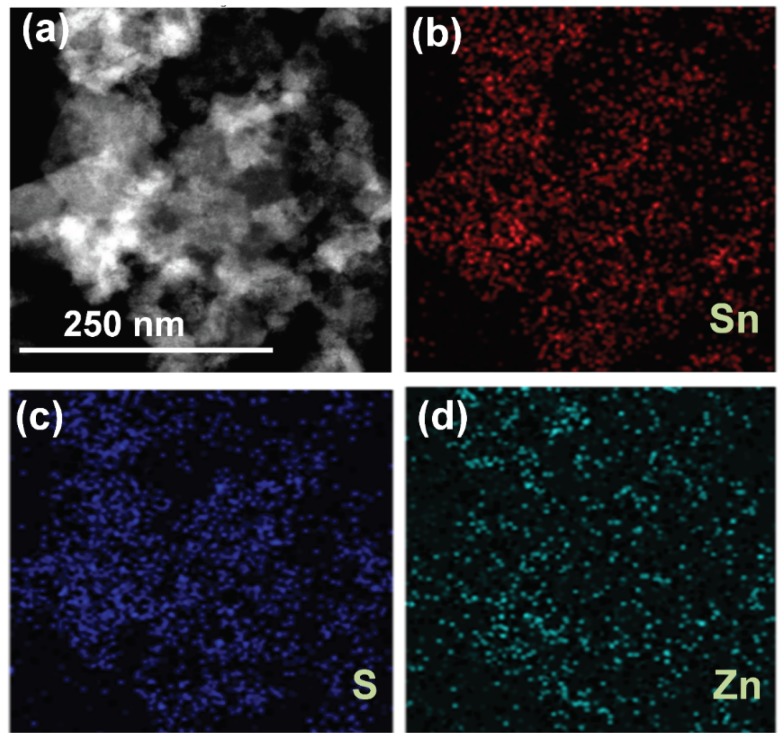
(**a**) TEM image of Sn_0.97_Zn_0.03_S_2_ nanoflakes and Energy dispersive spectroscopy (EDS) elemental mapping of Sn (**b**), S (**c**) and Zn (**d**) from selected area for 2D Sn_0.97_Zn_0.03_S_2_.

The morphological features of SnS_2,_ Sn_0.99_Zn_0.01_S_2_ (Figure S1) and Sn_0.97_Zn_0.03_S_2_ products were examined with the aid of field-emission scanning electron microscope (FESEM) technique. The image seen from Figure 1a–c confirms hexagonal nanoflakes with smooth surface and homogeneous distribution in case of pristine SnS_2_. However, on doping with Zinc the morphology appears to be similar with that of pristine nanoflakes with some random aggregates on the surface of SnS_2_ (Figure 1d,e). Additionally, transmission electron microscope (TEM) was employed to further investigate the detailed morphological information of SnS_2_ and Sn_0.97_Zn_0.03_S_2_ products. Figure 2 shows TEM images of pristine SnS_2_ and Sn_0.97_Zn_0.03_S_2_ nanoflakes with different magnifications. From the Figure 2a–c, it is clear that pristine SnS_2_ possess typical nanoflakes like structures with hexagonal stacking. Similarly the Sn_0.97_Zn_0.03_S_2_ nanoflakes (Figure 2d–f) also possess indistinguishable hexagonal morphology of pristine SnS_2_. The inset of Figure 2c,f displays the selected area electron diffraction (SAED) pattern revealing polycrystalline structure of the obtained samples. Energy dispersive spectroscopy (EDS) analysis was further employed in TEM mode to study the homogeneous distribution of Zn element in Sn_0.97_Zn_0.03_S_2_ nanoflakes. Figure 3a–d displays the TEM image and TEM-EDS mapping of Sn_0.97_Zn_0.03_S_2_ nanoflakes. As seen from Figure 3d, Zn element is distributed evenly throughout the whole structure of Sn_0.97_Zn_0.03_S_2_ nanoflakes.

**Figure 4:**
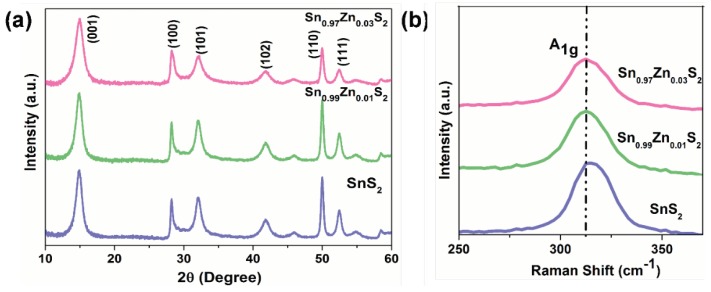
Structure properties of SnS_2_, Sn_0.99_Zn_0.01_S_2_ and Sn_0.97_Zn_0.03_S_2_ nanoflakes. (**a**) X-ray diffraction pattern of SnS_2_, Sn_0.99_Zn_0.01_S_2_ and Sn_0.97_Zn_0.03_S_2_ nanoflakes; (**b**) Raman spectrum of SnS_2_, Sn_0.99_Zn_0.01_S_2_ and Sn_0.97_Zn_0.03_S_2_ nanoflakes at excitation wavelength of 532 nm.

The crystallographic pattern of as synthesized SnS_2_, Sn_0.99_Zn_0.01_S_2_ and Sn_0.97_Zn_0.03_S_2_ nanoflakes are investigated by XRD analysis and presented in Figure 4a. Here, the strong diffraction peak observed at 2θ = 14.92° belongs to (001) diffraction, is an indication of the hexagonal structure of SnS_2_ [37]. However, the diffraction peak (001) tends to shift towards smaller angle on Zn doping. This shifting indicates that Zn ions replace Sn sites in the SnS_2_ crystal matrix. Furthermore, no peaks related to other compounds namely, ZnS and ZnSnS_3_ are observed in the XRD pattern. Additionally, Raman measurement was further analyzed to study detailed information about the structural properties of Zn doped SnS_2_ nanoflakes. Raman spectrum for sample SnS_2,_ Sn_0.99_Zn_0.01_S_2_ and Sn_0.97_Zn_0.03_S_2_ nanoflakes are displayed in Figure 4b. Here, in case of pristine SnS_2_, Sn_0.99_Zn_0.01_S_2_ and Sn_0.97_Zn_0.03_S_2_ nanoflakes, a strong signal was observed at 312 cm^−1^, which is related to A_1g_ phonon vibration mode of SnS_2_ [38,39,40].

**Figure 5:**
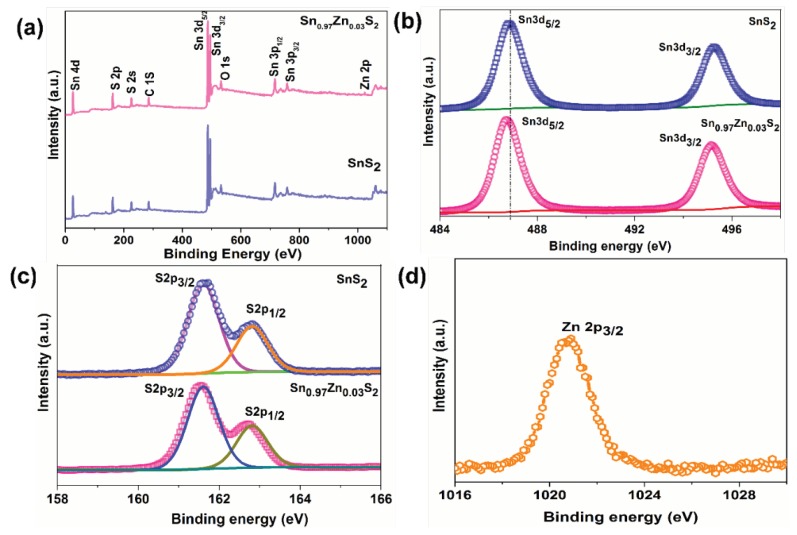
(**a**) Full survey spectra of SnS_2_ and Sn_0.97_Zn_0.03_S_2_ sample. (**b**) X-ray photoelectron spectroscopy (XPS) core level Sn 3d spectra of SnS_2_ and Sn_0.97_Zn_0.03_S_2_ nanoflakes. (**c**) S 2p core level spectra of SnS_2_ and Sn_0.97_Zn_0.03_S_2_ nanoflakes. (**d**) Zn 2p core level spectra of Sn_0.97_Zn_0.03_S_2_.

To elucidate the chemical composition of pristine and Sn_0.97_Zn_0.03_S_2_ nanoflakes, XPS measurements have been carried out and shown in Figure 5a. XPS full survey spectrum (Figure 5a) confirms the presence of Zn doping in SnS_2_. Figure 5b,c displays the XPS spectra of Sn 3d and S 2p peaks for Sn_0.97_Zn_0.03_S_2_ nanoflakes. As observed in Figure 5b,c, the peaks of Sn 3d at 486.33 and 494.4 eV of Sn 3d is ascribed to Sn3d_3/2_ and Sn3d_5/2_ and peaks at 161.2 and 163.3 eV correspond to S 2p peaks of SnS_2_. These results are consistent with those reported for SnS_2_ [41,42]. The binding energies of Sn 3d_5/2_ peak corresponding to pristine SnS_2_ was observed at 486.47 eV. Subsequently doping with Zn on SnS_2_, peaks of Sn 3d_5/2_ shifts to lower energy position to 486.33 eV. The shifting in the binding energy value of Sn 3d_5/2_ peak was about 0.14 eV compared to pristine SnS_2_. This shift might be due to Zn ion replace Sn sites in the SnS_2_ crystal lattice. Figure 5d shows the XPS spectrum for Zn in SnS_2_ nanoflakes. Besides, the Zn 2p_3/2_ peak appeared at 1021.3 eV is attributed to Zn^2+^ bonding state [43], confirming Zn^2+^ ions have been incorporated into the SnS_2_.

**Figure 6:**
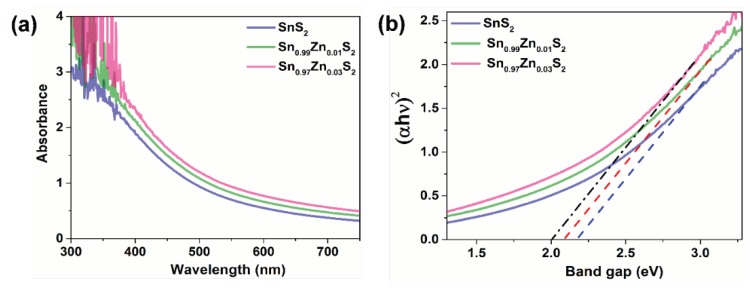
Properties of SnS_2_, Sn_0.99_Zn_0.01_S_2_ and Sn_0.97_Zn_0.03_S_2_ nanoflakes. (**a**) UV−vis absorption spectrum of the SnS_2_, Sn_0.99_Zn_0.01_S_2_ and Sn_0.97_Zn_0.03_S_2_ nanoflakes. (**b**) Tauc’s plot extracted from the absorption spectrum revealing their direct band gap.

Figure 6a shows UV–visible absorption spectrum of SnS_2_, Sn_0.99_Zn_0.01_S_2_ and Sn_0.97_Zn_0.03_S_2_ in the range of 300–750 nm. SnS_2_ displays a strong absorption in visible part of the solar spectrum. However, in contrast the samples Sn_0.99_Zn_0.01_S_2_ and Sn_0.97_Zn_0.03_S_2_ displayed a broad light absorption in 300 to 750 nm, which indicates that doping Zn ion can result in extending of absorption edge of SnS_2_. This results suggests that samples Sn_0.97_Zn_0.03_S_2_ possess greater potential than that of pristine sample SnS_2_ to drive photo excited charge carriers under the light irradiation. The values estimated was found to be 2.24 eV for sample SnS_2_ which is consistent with our previous result (Figure 6b). However, the values was found to be 2.19 and 2.09 eV for sample Sn_0.99_Zn_0.01_S_2_ and Sn_0.97_Zn_0.03_S_2_. It shows band gap becomes narrower than pristine SnS_2_ as the Zn content increases [44,45]. This reduction in the band gap might be due to modification in the electronic structures of SnS_2_ due to Zn doping, which results in creating energy levels in the band gap. This band gap could result in better absorption in visible region and can increase photo excited charge carriers under illumination.

Mott–Schottky (M–S) analysis was made to study the electrical properties of pristine SnS_2_, Sn_0.99_Zn_0.01_S_2_ and Sn_0.97_Zn_0.03_S_2_ nanoflakes. Generally, Mott–Schottky plot was employed to determine the donor density (*N*_d_) and flat band potential (*V*_fb_) of the materials. M–S analysis are generally expressed by [46,47,48]

**Figure 7:**
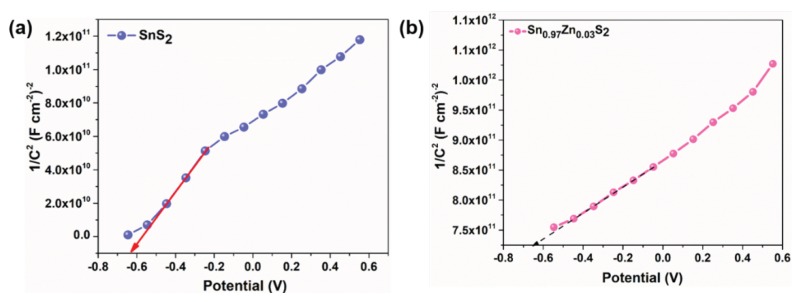
Mott–Schottky plots of (**a**) SnS_2_ and (**b**) Sn_0.97_Zn_0.03_S_2_ nanoflakes.

1/*C*^2^ = (2/eεε*_o_N*_d_)[(*V*_fb_ − *V*) − k_B_*T*/e] (1)

where e is the electronic charge, ε is the dielectric constant of SnS_2_, ε_0_ is the relative permittivity, *N*_d_ dopant density, *V* the applied potential, *C* the specific capacitance, k_B_ the Boltzmann constant and *V*_fb_ the flat band potential. The M–S plots of pristine SnS_2_, Sn_0.99_Zn_0.01_S_2_ (Figure S2) and Sn_0.97_Zn_0.03_S_2_ nanoflakes are displayed in Figure 7. Here *V*_fb_ was determined from intercept between the extrapolated linear plot of the curve and was estimated to be ~0.67 V for pristine SnS_2_ and 0.64 V for Sn_0.97_Zn_0.03_S_2_ nanoflakes. Additionally the difference in the slope reflects the variation in the carrier density (*N*_d_). The values of carrier density was estimated from the Equation (1) to be about 1.46 × 10^19^ and 0.47 × 10^19^ and in case of SnS_2_ and Sn_0.97_Zn_0.03_S_2_ nanoflakes.

**Figure 8:**
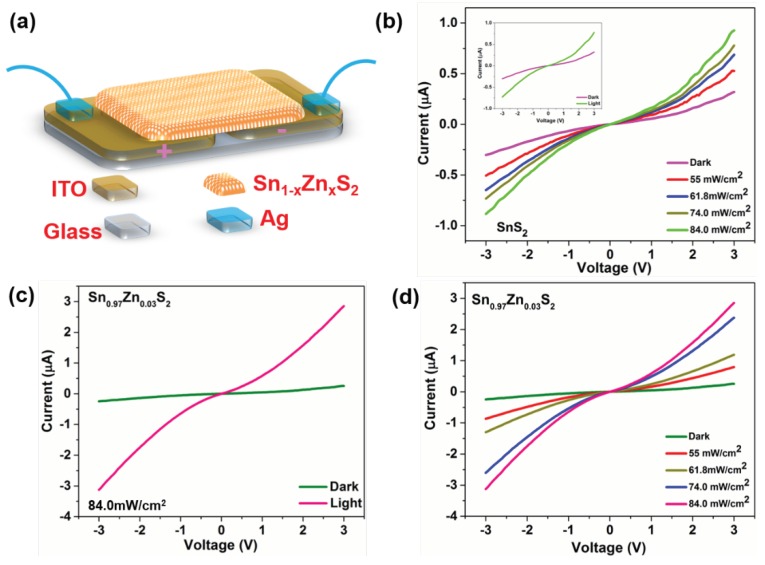
(**a**) Schematic representation of the photoelectronic device. (**b**) I-V characteristics of SnS_2_ device under different illumination intensities (Inset shows the I-V characteristics under dark and illumination intensity 84.0 mW/cm^2^). (**c**) I-V characteristics of Sn_0.97_Zn_0.03_S_2_ device under illumination conditions. (**d**) I-V characteristics of Sn_0.97_Zn_0.03_S_2_ device under different light intensities (55, 61.8, 74.0, 84.0 mW/cm^2^).

A photoelectronic device was constructed on samples SnS_2_ and Sn_0.97_Zn_0.03_S_2_ to study its potential for optoelectronics applications (Figure 8a), (for the details of fabrication process refer Expt. sections). I-V curves of pristine SnS_2_ nanoflakes at various illumination intensities and dark condition is displayed in Figure 8b. Inset shows I-V curves of the pristine SnS_2_ nanoflakes under dark and illumination. Here, the I-V curve shows a roughly symmetric behavior indicating Schottky-like junction established at ITO and SnS_2_ contacts. The dark current was noted to be 0.29 μA at a bias of 3 V. In contrast, the enhancement of current was measured and the value reaches to 0.98 μA under illumination, demonstrating excellent photosensitivity of the SnS_2_ samples. I-V curves of Sn_0.97_Zn_0.03_S_2_ nanoflakes device under illumination and dark is displayed in Figure 8c. Here, the value of dark current was found to increase than that of pristine SnS_2_, which suggests reduction in resistance of SnS_2_ after Zn doping. However, a notable enhancement in photocurrent under illumination was noted compared to that of dark current at same bias voltage in Sn_0.97_Zn_0.03_S_2_ nanoflakes device, indicating their excellent sensitivity. Moreover, photo to dark current (*I*_light_/*I*_dark_) ratio for Sn_0.97_Zn_0.03_S_2_ device (~10.1) tends to increase compared to pristine SnS_2_ (~3.37). The high sensitivity and enhancement in photocurrent of Sn_0.97_Zn_0.03_S_2_ nanoflakes reveal the effective separation of photoexcited carriers in samples, which are actually promoted after Zn-doping. Figure 8d shows I-V curves of the Sn_0.97_Zn_0.03_S_2_ device measured at room temperature under different light intensities. The photocurrent increases with increasing light intensities revealing strong and clear photon-induced currents phenomena, indicating excellent photoresponse ability of the device. Under illumination, photoexcited charge carriers are mainly generated in Sn_0.97_Zn_0.03_S_2_. Then the charge carriers are quickly separated and driven towards the nearby electrodes due to built-in electric field created at the interface, resulting in photocurrent generation.

**Figure 9:**
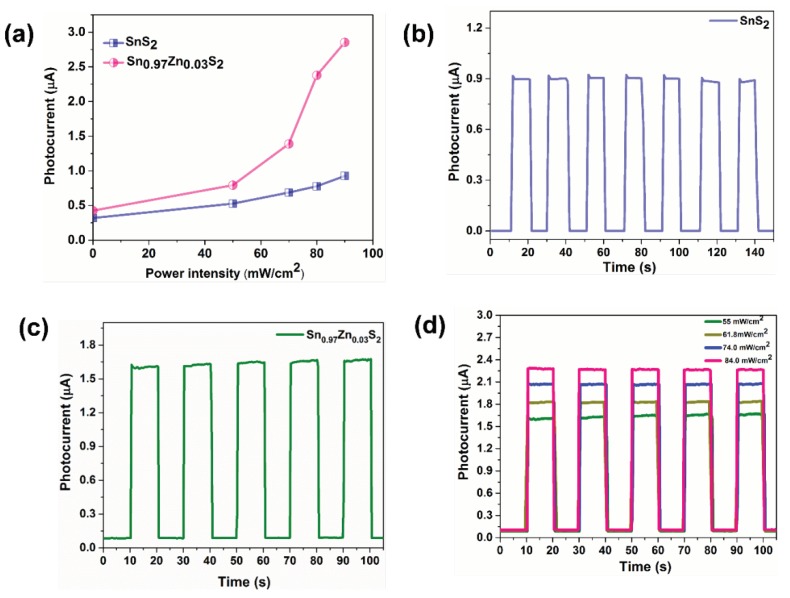
(**a**) Light intensity-dependent photocurrent values of pristine SnS_2_ and Sn_0.97_Zn_0.03_S_2_ device. Time-dependent photocurrent response of (**b**) SnS_2_ device and (**c**) Sn_0.97_Zn_0.03_S_2_. (**d**) Time-dependent photocurrent response of Sn_0.97_Zn_0.03_S_2_ device under different illumination intensities.

Figure 9a shows light intensity-dependent photocurrent values of pristine SnS_2_ and Sn_0.97_Zn_0.03_S_2_ device. The observed photocurrent value to illumination intensities suggest that the charge carrier photo-generation efficiency is proportional to the number of photons absorbed by the pristine SnS_2_ and Sn_0.97_Zn_0.03_S_2_ nanoflakes. Reliable response speed and stability to illumination conditions are crucial for the photoelectronic device. To address this concern, time related photoresponse of pristine SnS_2_ and Sn_0.97_Zn_0.03_S_2_ device was measured with turning light on/off condition for a period of 10 seconds for multiple cycles. Figure 9b,c shows time related photoresponse of the pristine and Sn_0.97_Zn_0.03_S_2_ device under several switch on and switch off conditions. Here, the photocurrent of pristine SnS_2_ was found to be 0.8 µA. Interestingly the photocurrent is improved by two fold in case of Sn_0.97_Zn_0.03_S_2_ nanoflakes (1.75 µA) compared to pristine SnS_2_ (Figure 9c). The photoresponse enhancement could be related to Zn ions which acts as an effective dopant and enhance charge separation taking place at the interface. The rise/decay time was measured to be 0.2 and 0.2 s. The reason for the relative longer response speed in our case is probably related to the formation of interface states between the Sn_0.97_Zn_0.03_S_2_ nanoflakes and ITO substrate, which can block the photo-generated carriers, resulting in long life time of the photo-generated carriers. Meanwhile, the device shows no fluctuation under illumination for several repetitive cycles, inferring the excellent stability of the Sn_0.97_Zn_0.03_S_2_ device. The time related response of the Sn_0.97_Zn_0.03_S_2_ device under varied light intensities are displayed in Figure 9d. Here, the photocurrent value varies with different light intensities demonstrating excellent reproducibility of Sn_0.97_Zn_0.03_S_2_ based device. Such high and stable photoresponse behavior may come from the fact that Zn ions act as an effective dopant and result in increased light absorption, which enhances photogenerated charge carriers and leads to an enhanced photocurrent of the device. Thus, photoelectrical studies on Sn_0.97_Zn_0.03_S_2_ nanoflakes illustrates that Zn doping in SnS_2_ results in significant enhancement of their optoelectronic properties, which leads to improved conductivity and sensitivity. 

**Figure 10:**
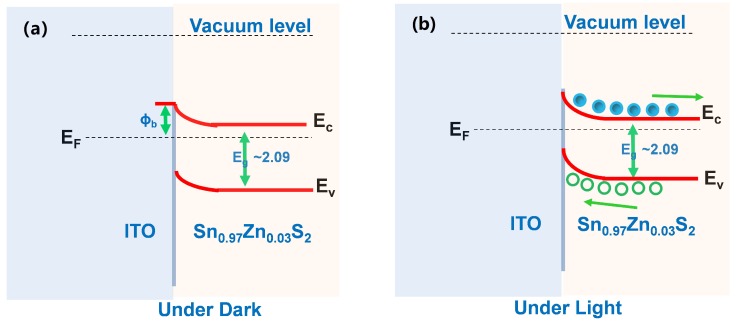
Energy diagram of the Sn_0.97_Zn_0.03_S_2_/ITO Schottky junction under (**a**) dark and (**b**) illumination conditions.

The mechanism involved in the enhanced photoresponse of Sn_0.97_Zn_0.03_S_2_/ITO structure was explained through energy band diagram in Figure 10. Since the work function between ITO and Sn_0.97_Zn_0.03_S_2_ is different, a Schottky-type behavior is established at Sn_0.97_Zn_0.03_S_2_/ITO interface (Figure 10). Due to this behavior, an electric field was established at the Sn_0.97_Zn_0.03_S_2_/ITO interface. This electric field then accelerates the separation of the photoexcited charge carriers without the application of any applied bias. When illuminated, photoexcited charge carriers produced in Sn_0.97_Zn_0.03_S_2_ are then separated at the Sn_0.97_Zn_0.03_S_2_/ITO interface. This charge carriers separation which was induced due to the electric field results in band bending at the Sn_0.97_Zn_0.03_S_2_/ITO interface. As a result, the photoexcited charge carriers are swept towards ITO electrodes, involving in enhancement of photocurrent (Figure 10b).

## 4. Conclusions

In summary, Sn_0.97_Zn_0.03_S_2_ nanoflakes were prepared via low temperature hydrothermal synthesis. The modulation of the structural and photoelectrical properties in SnS_2_ via doping Zinc have been discussed in detail. A shift in XPS peak of Sn 3d_5/2_ and S 2p_3/2_ has been observed in Sn_0.97_Zn_0.03_S_2_ nanoflakes due to Zn ion replaced Sn sites in the SnS_2_ crystal lattice. Optical properties studies show that Sn_0.97_Zn_0.03_S_2_ nanoflakes possess higher visible-light absorption than that of pristine SnS_2_. Photoelectrical properties based on Sn_0.97_Zn_0.03_S_2_ nanoflakes reveal that Zn doping leads to significant improvement in conductivity and sensitivity to illuminations compared to pristine SnS_2_. Such an excellent performance of Sn_0.97_Zn_0.03_S_2_ nanoflakes may endow it as a potential candidate for emerging 2D materials in optoelectronic applications.

## Supplementary Materials

The following are available online at https://www.mdpi.com/2079-4991/9/7/924/s1, Figure S1: SEM image of Sn_0.99_Zn_0.01_S_2_ nanoflakes, Figure S2: Mott–Schottky plot of Sn_0.99_Zn_0.01_S_2_ nanoflakes.

## References

[1] Wang Q.H., Kalantar-Zadeh K., Kis A., Coleman J.N., Strano M.S. (2012). Electronics and optoelectronics of two-dimensional transition metal dichalcogenides. Nat. Nanotechnol..

[2] Najmaei S., Liu Z., Zhou W., Zou X., Shi G., Lei S., Yakobson B.I., Idrobo J., Ajayan P.M., Lou J. (2013). Vapour phase growth and grain boundary structure of molybdenum disulphide atomic layers. Nat. Mater..

[3] Chhowalla M., Shin H.S., Eda G., Li L.-J., Loh K.P., Zhang H. (2013). The Chemistry of two- dimensional layered transition metal dichalcogenides nanoflakes. Nat. Chem..

[4] Van der Zande A.M., Huang P.Y., Chenet D.A., Berkelbach T.C., You Y., Lee G.-H., Heinz T.F., Reichman D.R., Muller D.A., Hone J.C. (2013). Grains and grain boundaries in highly crystalline monolayer molybdenum disulphide. Nat. Mater..

[5] Cheng L., Huang W., Gong Q., Liu C., Liu Z., Li Y., Dai H. (2014). Ultrathin WS_2_ nanoflakes as a high-performance electrocatalyst for the hydrogen evolution reaction. Angew. Chem. Int. Ed..

[6] Peimyoo N., Yang W., Shang J., Shen X., Wang Y., Yu T. (2014). Chemically driven tunable light emission of charged and neutral excitons in monolayer WS_2_. ACS Nano.

[7] Late D.J., Liu B., Ramakrishna Matte H.S.S., Dravid V.P., Rao C.N.R. (2012). Hysteresis in single-layer MoS_2_ field effect transistors. ACS Nano.

[8] Wang H., Yuan H., Hong S.S., Li Y., Cui Y. (2015). Physical and chemical tuning of two-dimensional transition metal dichalcogenides. Chem. Soc. Rev..

[9] Sun Y., Sun Z., Gao S., Cheng H., Liu Q., Piao J., Yao T., Wu C., Hu S., Wei S. (2012). Fabrication of flexible and freestanding zinc chalcogenide single layers. Nat. Commun..

[10] Lei F., Sun Y., Liu K., Gao S., Liang L., Pan B., Xie Y. (2014). Oxygen vacancies confined in ultrathin indium oxide porous sheets for promoted visible-light water splitting. J. Am. Chem. Soc..

[11] Yang J., Son J.S., Yu J.H., Joo J., Hyeon T. (2013). Advances in the colloidal synthesis of two-dimensional semiconductor nanoribbons. Chem. Mater..

[12] Huang J.-K., Pu J., Hsu C.-L., Chiu M.-H., Juang Z.-Y., Chang Y.-H., Chang W.-H., Iwasa Y., Takenobu T., Li L.-J. (2014). Large area synthesis of highly crystalline WSe_2_ monolayers and device applications. ACS Nano.

[13] Zeng Z., Yin Z., Huang X., Li H., He Q., Lu G., Boey F., Zhang H. (2011). Single-layer semiconducting nanoflakes: High-yield preparation and device fabrication. Angew. Chem. Int. Ed..

[14] Xia J., Zhu D., Wang L., Huang B., Huang X., Meng X.-M. (2015). Large-scale growth of two-dimensional SnS_2_ crystals driven by screw dislocations and application to photodetectors. Adv. Funct. Mater..

[15] Fu X., Ilanchezhiyan P., Mohan Kumar G., Cho H.D., Zhang L., Sattar Chan A., Lee D.J., Panin G.N., Kang T.W. (2017). Tunable UV-visible absorption of SnS_2_ layered quantum dots produced by liquid phase exfoliation. Nanoscale.

[16] Tao Y., Wu X., Wang W., Wang J. (2015). Flexible photodetector from ultraviolet to near infrared based on a SnS_2_ nanosheet microsphere film. J. Mater. Chem. C.

[17] Huang Y., Deng H.-X., Xu K., Wang Z.-X., Wang Q.-S., Wang F.-M., Wang F., Zhan X.-Y., Li S.-S., Luo J.-W. (2015). Highly sensitive and fast phototransistor based on large size CVD-grown SnS_2_ nanoflakes. Nanoscale.

[18] Mohan Kumar G., Fu X., Ilanchezhiyan P., Yuldashev S.U., Lee D.J., Cho H.D., Kang T.W. (2017). Highly sensitive flexible photodetectors based on self-assembled tin monosulfide nanoflakes with graphene electrodes. ACS Appl. Mater. Interfaces.

[19] Mohan Kumar G., Xiao F., Ilanchezhiyan P., Yuldashev S.U., Kang T.W. (2016). Enhanced photoelectrical performance of chemically processed SnS_2_ nanoplates. RSC Adv..

[20] De D., Manongdo J., See S., Zhang V., Guloy A., Peng H.B. (2013). High on/off ratio field effect transistors based on exfoliated crystalline SnS_2_ nano-membrane. Nanotechnology.

[21] Ye X., Chen J., Engel M., Millan J.A., Li W., Qi L., Xing G., Collins J.E., Kagan C.R., Li J. (2013). Competition of shape and interaction patchiness for self-assembling nanoplates. Nat. Chem..

[22] Ye X., Collins J.E., Kang Y., Chen J., Chen D.T., Yodh A.G., Murray C.B. (2010). Morphologically controlled synthesis of colloidal upconversion nanophosphors and their shape-directed self-assembly. Proc. Natl. Acad. Sci. USA.

[23] Novoselov K.S., Fal’ko V.I., Colombo L., Gellert P.R., Schwab M.G., Kim K. (2012). A roadmap for graphene. Nature.

[24] Zhang K., Zhang T.N., Cheng G.H., Li T.X., Wang S.X., Wei W., Zhou X.H., Yu W.W., Sun Y., Wang P. (2016). Interlayer transition and infrared photodetection in atomically thin type-II MoTe_2_/MoS_2_ van der Waals heterostructures. ACS Nano.

[25] Zhang Y., Zhu P., Huang L., Xie J., Zhang S., Cao G., Zhao X. (2015). Few-layered SnS_2_ on few-layered reduced graphene oxide as Na-Ion battery anode with ultralong cycle life and superior rate capability. Adv. Funct. Mater..

[26] Tan F.R., Qu S.C., Wu J., Liu K., Zhou S.Y., Wang Z.G. (2011). Preparation of SnS_2_ colloidal quantum dots and their application in organic/inorganic hybrid solar cells. Nanoscale Res. Lett..

[27] Su G., Hadjiev V.G., Loya P.E., Zhang J., Lei S., Maharjan S., Dong P., Ajayan P.M., Lou J., Peng H. (2015). Chemical vapor deposition of thin crystals of layered semiconductor SnS_2_ for fast photodetection application. Nano Lett..

[28] Zhou X., Zhang Q., Gan L., Li H., Zhai T. (2016). Large-size growth of ultrathin SnS_2_ nanoflakes and high performance for phototransistors. Adv. Funct. Mater..

[29] Wei R., Hu J., Zhou T., Zhou X., Liu J., Li J. (2014). Ultrathin SnS_2_ nanoflakes with exposed {001} facets and enhanced photocatalytic properties. Acta Mater..

[30] Wang J., Liu J., Xu H., Ji S., Wang J., Zhou Y., Hodgson P., Li Y. (2013). Gram-scale and template- free synthesis of ultralong tin disulfide nanobelts and their lithium ion storage performances. J. Mater. Chem. A.

[31] Cui X., Xu W., Xie Z., Dorman J.A., Gutierrez-Wing M.T., Wang Y. (2016). Effect of dopant concentration on visible light driven photocatalytic activity of Sn_1−*x*_Ag*_x_*S_2_. Dalton Trans..

[32] Wahnón P., Conesa J.C., Palacios P., Lucena R., Aguilera I., Seminovski Y., Fresno F. (2011). V-doped SnS_2_: A new intermediate band material for a better use of the solar spectrum. Phys. Chem. Chem. Phys..

[33] Hu K., Wang D., Zhao W., Gu Y., Bu K., Pan J., Qin P., Zhang X., Huang F. (2018). Intermediate band material of titanium-doped tin disulfide for wide spectrum solar absorption. Inorg. Chem..

[34] Li B., Xing T., Zhong M., Huang L., Lei N., Zhang J., Li J., Wei Z. (2017). A two-dimensional Fe-doped SnS_2_ magnetic semiconductor. Nat. Commun..

[35] Mohan Kumar G., Fu X., Ilanchezhiyan P., Yuldashev S.U., Madhan Kumar A., Cho H.D., Kang T.W. (2018). High performance photodiodes based on chemically processed Cu doped SnS_2_ nanoflakes. Appl. Surf. Sci..

[36] Liu J., Liu X., Chen Z., Miao L., Liu X., Li B., Tang L., Chen K., Liu Y., Li J. (2018). Tunable Schottky barrier width and enormously enhanced photoresponsivity in Sb doped SnS_2_ monolayer. Nano Res..

[37] Yu J., Xu C.-Y., Ma F.-X., Hu S.-P., Zhang Y.-W., Zhen L. (2014). Monodisperse SnS_2_ nanoflakes for high-performance photocatalytic hydrogen generation. ACS Appl. Mater. Interfaces.

[38] Qu B., Ma C., Ji G., Xu C., Xu J., Meng Y.S., Wang T., Lee J.Y. (2014). Layered SnS_2_-reduced graphene oxide composite—a high-capacity, high-rate, and long-cycle life sodium- ion battery anode material. Adv. Mater..

[39] Du Y., Yin Z., Rui X., Zeng Z., Wu X.J., Liu J., Zhu Y., Zhu J., Huang X., Yan Q. (2013). A facile, relative green, and inexpensive synthetic approach toward large-scale production of SnS_2_ nanoplates for high-performance lithium-ion batteries. Nanoscale.

[40] Chen Q., Lu F., Xia Y., Wang H., Kuang X. (2017). Interlayer expansion of few layered Mo-doped SnS_2_ nanoflakes grown on carbon cloth with excellent lithium storage performance for lithium ion batteries. J. Mater. Chem. A.

[41] Liu X., Zhao H.L., Kulka A., Trenczek-Zajac A., Xie J.Y., Chen N., Swierczek K. (2015). Characterization of the physicochemical properties of novel SnS_2_ with cubic structure and diamond-like Sn sublattice. Acta Mater..

[42] Ilanchezhiyan P., Kumar G.M., Kang T.W. (2015). Electrochemical studies of spherically clustered MoS_2_ nanostructures for electrode applications. J. Alloys Compd..

[43] Liu X., Bai H. (2013). Hydrothermal synthesis of visible light active zinc-doped tin disulfide photocatalyst for the reduction of aqueous Cr(VI). Powder Technol..

[44] An X., Yu J.C., Tang J. (2014). Biomolecule-assisted fabrication of Copper doped SnS_2_ nanosheet–reduced graphene oxide junctions with enhanced visible-light photocatalytic activity. J. Mater. Chem. A.

[45] Yassin O.A., Abdelaziz A.A., Jaber A.Y. (2015). Structural and optical characterization of V-and W-doped SnS_2_ thin films prepared by spray pyrolysis. Mater. Sci. Semicond. Process..

[46] Patel M., Chavda A., Mukhopadhyay I., Kim J., Ray A. (2016). Nanostructured SnS with inherent anisotropic optical properties for high photoactivity. Nanoscale.

[47] Wang L., Xia L., Wu Y., Tian Y. (2016). Zr-Doped β-In_2_S_3_ ultrathin nanoflakes as photoanodes: Enhanced visible-light-driven photoelectrochemical water splitting. ACS Sustain. Chem. Eng..

[48] Mohan Kumar G., Ilanchezhiyan P., Madhan Kumar A., Yuldashev S.U., Kang T.W. (2016). Electrical property studies on chemically processed polypyrolle/aluminum doped ZnO based hybrid heterostructures. Chem. Phys. Lett..

