# Supplementary material for: Effective Modulation of Optical and Photoelectrical Properties of SnS2 Hexagonal Nanoflakes via Zn Incorporation
Source: Nanomaterials (Basel). 2019 Jun 27;9(7):924. doi: 10.3390/nano9070924 (PMC6669736; doi:10.3390/nano9070924)
Supplement: Supplementary file 1 [file nanomaterials-09-00924-s001.pdf]

Supporting Information

Article

Effective Modulation of Optical and Photoelectrical Properties of SnS_2 Hexagonal Nanoflakes via Zn Incorporation

Ganesan Mohan Kumar [1], Pugazhendi Ilanchezhiyan [1,*], Hak Dong Cho [2], Shavkat Yuldashev [1], Hee Chang Jeon [2], Deuk Young Kim [3] and Tae Won Kang [1]

1 Nano-Information Technology Academy (NITA), Dongguk University-Seoul, 04623 Seoul, Korea; gmohankumar@dongguk.edu (G.M.K.); shavkat@dongguk.edu (Sh.Y.); twkang@dongguk.edu (T.W.K.)
2 Quantum Functional Semiconductor Research Center, Dongguk University-Seoul, 04623 Seoul, Korea; hakcho1@hotmail.com (H.D.C.); hcjeon@dongguk.edu (H.C.J.)
3 Division of Physics and Semiconductor Science, Dongguk University-Seoul, 04623 Seoul, Korea; dykim@dgu.edu
* Correspondence: ilancheziyan@dongguk.edu

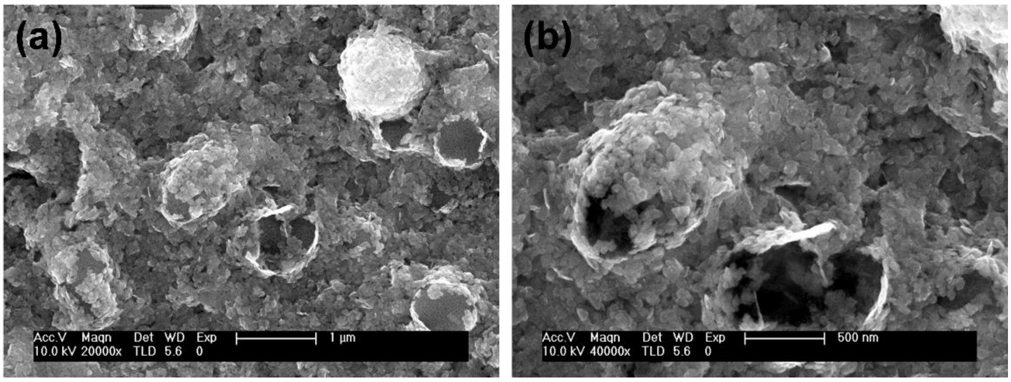

Figure S1. (**a**) Low magnification SEM image of $Sn_{0.99}Zn_{0.01}S_2$ nanoflakes. (**b**) High magnification SEM image of $Sn_{0.99}Zn_{0.01}S_2$ nanoflakes showing their hexagonal structure.

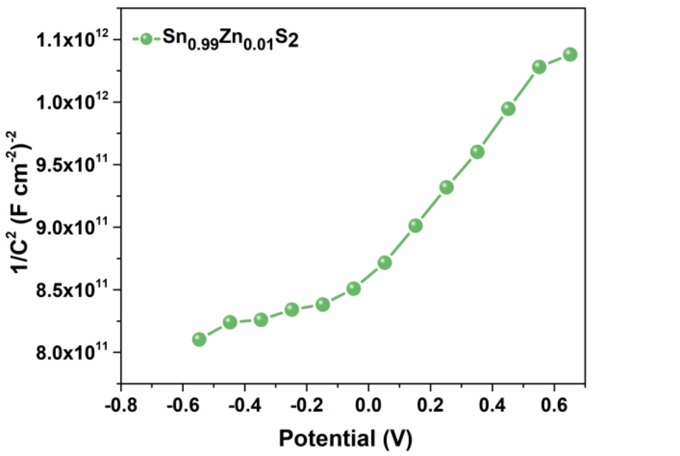

Figure S2. Mott-Schottky plot of $Sn_{0.99}Zn_{0.01}S_2$ nanoflakes.
